# Supplementary material for: Anomalous resistivity upturn in epitaxial L21-Co2MnAl films
Source: Sci Rep. 2017 Feb 20;7:42931. doi: 10.1038/srep42931 (PMC5317171; doi:10.1038/srep42931)
Supplement: Supplementary Materials [file srep42931-s1.pdf]

Supplemental materials for

Anomalous resistivity upturn in epitaxial $L2_1$-Co_2MnAl films

L. J. Zhu[1,2 a)], J. H. Zhao[2]

[1] *Cornell University, Ithaca, NY 14850, USA*
[2] *State Key Laboratory of Superlattices and Microstructures, Institute of Semiconductors, Chinese Academy of Sciences, P. O. Box 912, Beijing 100083, China*

[a)] Author to whom correspondence should be addressed;
Electronic mail: zhulijun0@gmail.com

Figure S1 shows an example of cross-sectional high-resolution tunneling electron microscopy (TEM) images for the Co_2MnAl films grown on GaAs (001) by molecular beam epitaxy. In good agreement with RHEED and XRD patterns, the TEM images confirmed the epitaxial growth and single-crystalline texture of the $L2_1$-Co_2MnAl films. The (111) and (002) diffraction patterns can be clearly observed in the fast Fourier transform analysis with the electron beam parallel to [1 1 0] axis of the Co_2MnAl film. This, together with the results of XRD θ-2θ scans and φ scans for Co_2MnAl (111), further enhances the evidence for the $L2_1$ ordering of these films.

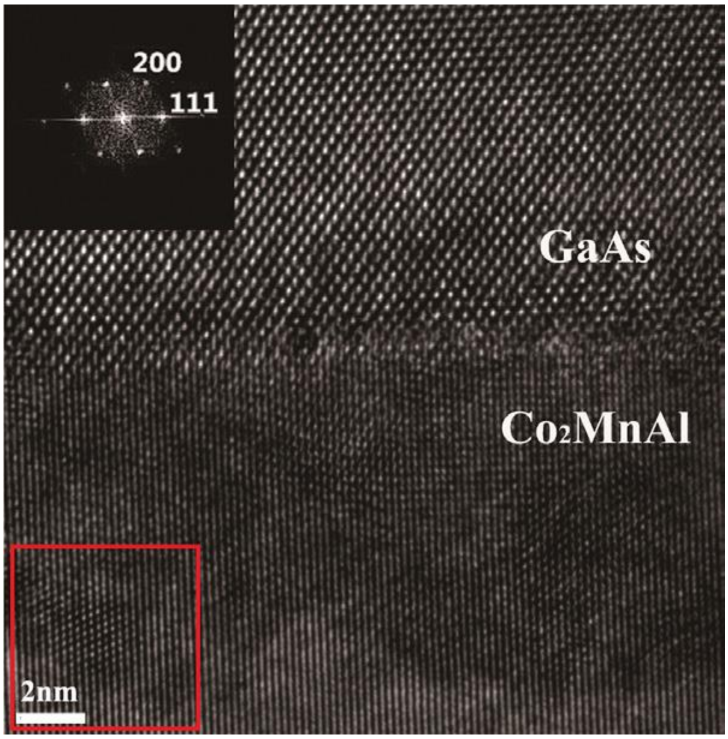

FIG.S1 A cross-sectional high-resolution TEM image of a Co_2MnAl film grown on GaAs. The inset shows the fast Fourier transform analysis performed on the area squared in the TEM image. Note that the electron beam is parallel to the [1 1 0] direction of the Co_2MnAl film.[5]

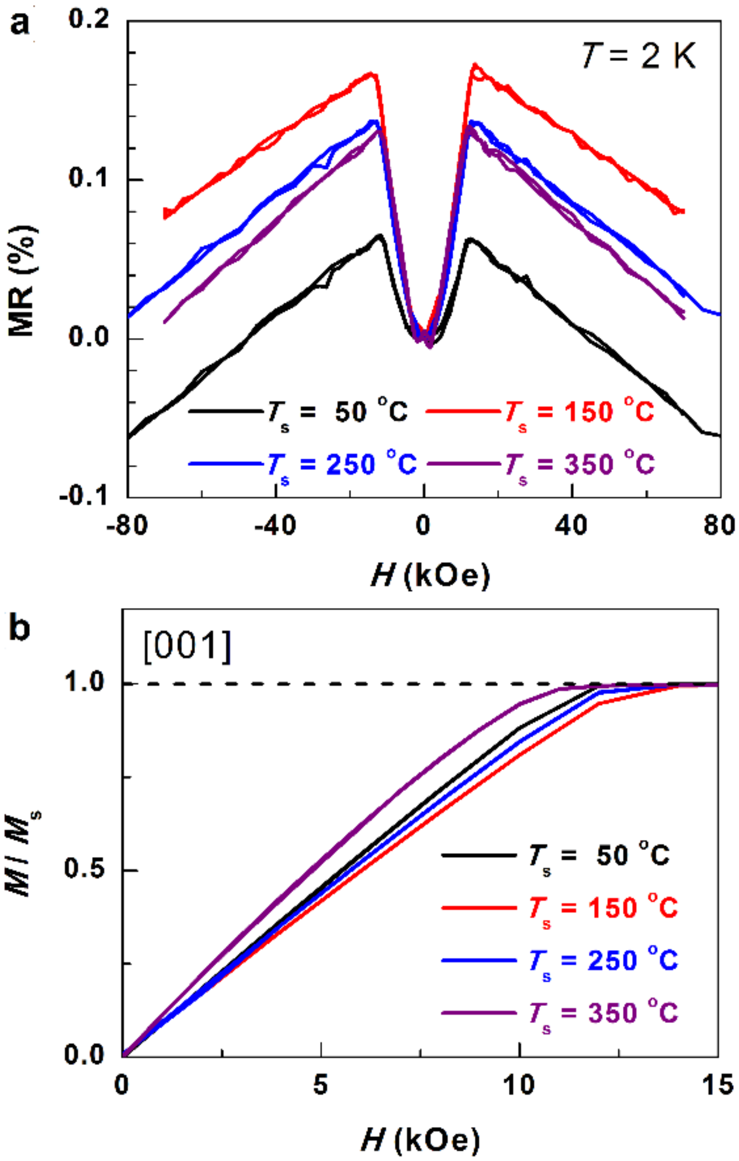

FIG. S2 (a) MR at 2 K and (b) Normalized magnetization hysteresis at 300 K along [001] direction for the $L2_1$-Co_2MnAl films with different T_s.

Figure S2(a) shows the magnetoresistance (MR) curves at 2 K for the $L2_1$-Co_2MnAl films with different T_s. In consistence with that at room temperature, each MR curve shows a dip at zero field and peaks at ~ ±15 kOe due to the anisotropic MR. The high-field MR which is linearly dependent on external magnetic field (*H*) also occurs at 2 K, excluding spin wave scattering as its source as magnons are unlikely to be excited at such low temperature. From Fig. S2(b), one can clearly see that all these films get saturated in magnetic moments along film normal when the applied fields exceeds 15 kOe, which is in consistence with the MR curves peaking at ~ ±15 kOe. This also indicates that the anisotropic MR should not be responsible for the high field MR that linearly scales with field.
